# Assessment of COVID-19 related preventive measures in medical students across a lower-middle-income country: A cross-sectional study from Pakistan

**DOI:** 10.1016/j.amsu.2022.104757

**Published:** 2022-09-25

**Authors:** Shoaib Ahmad, Sheza Hassan, Umar Farooq, Shkaib Ahmad, Sumera Ehsan, Daniyal Mansoor Ali, Mohammad Yasir Essar, Hamza Farooq Khan, Hashim Talib Hashim

**Affiliations:** a Punjab Medical College, Faisalabad, Pakistan; b Aga Khan University Medical College, Karachi, Pakistan; c Dera Ghazi Khan Medical College, Dera ghazi khan, Pakistan; d Health Education Department, Allied Hospital, Faisalabad, Pakistan; e Centre of Excellence Trauma and Emergencies, Aga Khan University, Karachi, Pakistan; f Kabul University of Medical Sciences, Kabul, Afghanistan; g College of Medicine, University of Baghdad, Iraq

**Keywords:** COVID-19, Cross-sectional study, Health behavior, Medical students, Preventive measures

## Abstract

**Objectives:**

This study aims to identify the effect of having COVID-19 positive close contact on practices and evaluate practices regarding COVID-19 prevention among medical students and the differences among clinical and preclinical students.

**Study design:**

The cross-sectional study included medical students from the Micro-fest++ event held on 30th May 2020.

**Methods:**

Participants filled a questionnaire of 15 questions regarding COVID preventive measures practices having satisfactory reliability (Cronbach's alpha = 0.715) and validity. The Statistical Package for Social Sciences (IBM SPSS) 26.0 was used for data analysis. Out of 1342 medical students, the majority were female (N = 881, 65.6%). Greater proportion (47%) of students had good practices (>85.7%) (p < 0.05).

**Results:**

Having COVID-19 positive relatives resulted in higher positive responses for practices with 11.86 ± 1.94 (out of 14) compared to 11.78 ± 2.38 for the COVID-19 negative group. Clinical year students compared to preclinical students responded positively to all questions, except one, and had a better score of 11.90 ± 2.28 (out of 14) compared to 11.61 ± 2.37 (p < 0.05). A significant difference was noted for “Information on preventive measures” (p < 0.01), “Avoiding crowds and staying home” (p < 0.05), “Social distancing (maintain 3 feet)" (p < 0.01), and “Practices of disinfection after going outside” (p < 0.05).

**Conclusions:**

Overall, medical students showed good practices, but a lack of knowledge in certain areas requires addressing infection during clinical rotations. A greater proportion of clinical students and those having a COVID-19 positive relative showed better adherence to practices.

## Highlights

• Having COVID-19 infected relatives had a positive impact on preventive measures.
• Clinical year students responded positively compared to preclinical ones.
• Gap of preventive practices was highly evident amongst both groups.

## 1 Introduction

In December 2019, an acute viral respiratory illness emerged in Wuhan, China [1]. Later in January 2020, due to its rapid spread, it was declared a public health emergency of international concern (PHEIC) by the World Health Organization (WHO). WHO named the disease Coronavirus disease 2019 (COVID-19) caused by SARS-CoV-2, a new strain of the coronavirus family.

COVID-19 has an extended incubation period of 2–14 days [2], with a clinical presentation varying from an asymptomatic course to a severe acute respiratory syndrome [3]. The most common transmission mode is respiratory droplets (coughing and sneezing) [4], contact with an infected person or contaminated surface, and transfer of the virus to the mouth, nose, or eyes [5]. Moreover, the most common clinical presentation of COVID-19 includes symptoms such as fever, anosmia, cough, and fatigue; chest imaging illustrating bilateral ground-glass appearance; and laboratory findings showing leukopenia and lymphopenia [6]. In severely affected individuals, the virus illness can result in progressive respiratory distress syndrome, shock, and even death [7]. Although SARS-CoV-2 has a faster transmission rate, the mortality rate is between 0.5 and 4.3%. To date, no specific antiviral treatment or vaccine has been developed for its prevention [8].

Pakistan reported its first case of COVID-19 on 26th February 2020. Since then, the virus has been spreading at an alarming rate. More than 435,000 confirmed cases had been reported, with the death toll being close to 9000. The most deaths reported are in Sindh (more than 2500) [9].

Medical students rotating in the wards are in direct contact with the patients and possible carriers. Lack of proper knowledge in this population can lead to increased infection rates, increased stress and anxiety levels, and alterations in applying their medical knowledge [10]. Therefore, this study's rationale was to assess the current practices among medical students regarding necessary protocols required for limiting the spread of infection and address the less practiced measures to prevent hospital infections. Moreover, this cross-sectional study also aimed to assess the difference in existing practices between clinical and preclinical students and whether having a COVID-19 positive family member or friend significantly impacted their prevention practices.

## 2 Methods

This cross-sectional study was conducted across medical colleges in Pakistan after approval from the Ethics Review Committee. Written consent was signed by the medical students before filling the form. No identifiable information was obtained, and each form was assigned an ID to keep the data and results of the study anonymous. This study has been conducted and is in line with STORCSS criteria [11]. Out of 1483 students who filled the questionnaire at the end of the national Micro-fest++ online quiz conducted on 30th May 2020, only 1342 completely attempted responses were selected based on the inclusion criteria with non-probability consecutive sampling. The study's inclusion criteria were; all medical students (≥18 years) who participated in the Micro-fest++ event and currently studying in any medical college across Pakistan. Participants from countries other than Pakistan were excluded.

Internal consistency for the questionnaire was calculated using Cronbach's alpha. A minimum reliability value of 0.7 is required for group comparison and 0.9 for individual assessment. For this study, alpha has a cumulative value of 0.715, making it fit for comparison across groups based on the received responses.

The data collected included information regarding demographics, gender, educational status, area of residence, and whether the participants belonged to the MBBS or BDS program. In the MBBS program, participants were asked which year of study (1–5) they were in. First and second year MBBS students were labeled as preclinical students, while 3rd to 5th years were categorized as clinical-year students. Moreover, the response was assessed based on whether they had a COVID-19 positive family member or not.

The questionnaire was designed according to the guidelines provided by WHO and by an epidemiologist's expert opinion [12]. For statistical analysis, IBM SPSS (Statistical Package for Social Sciences) Version 26.0 was used. Descriptive analysis was presented as mean ± SD and N (%) for continuous and categorical variables. To compare the mean scores of practices among the clinical and preclinical groups, an independent sample *t*-test was used. For assessing attitudes, the mean scores of practices were compared for those who had close COVID-19 positive patient with those who did not. A p-value < 0.05 was considered significant throughout.

## 3 Results

Out of 1483 students who filled the questionnaire at the end of the national Micro-fest++ online quiz conducted on 30th May 2020, 1342 complete responses were selected. The details of the study population's demographic characteristics are outlined in Table 1 (Supplementary Material).

The questionnaire's preclinical and clinical year students' responses are detailed in Table 2 (Supplementary Material). The percentage of “Yes” and “No” was calculated and tabulated for all the questions. P-value has been tabulated with every question.

This sample size had a confidence interval of 97.09% and an error margin of ±2.91 as calculated by the Rao-soft Inc. sample size calculator. The questionnaire was placed along with informed consent and associated terms and conditions. The questionnaire consisted of 14 questions inquiring about participants' practices about coronavirus, with one question reserved to inquire if they had a close COVID-19 patient to assess the variation in attitudes.

In study questionnaire, 14 questions addressed the practices of prevention. According to the results, more clinical year students responded positively than preclinical students in this category except for one variable (managing family stress). However, the response to Question (Information regarding preventive measures) and “Social distancing (maintain 3 feet distance)" had p < 0.01 while “Avoiding crowded areas and staying at home” and “Family practices of disinfection after coming back from outside” had a p-value of <0.05.

One last question was asked to acquire information about whether participants had a COVID-19 positive family member/friend or not.

For assessment of attitude change, having a COVID-19 positive family member or friend on the responses related to practices was also compared (Table 3, Supplementary Material). Out of 1342 participants, 211 (15.7%) had a COVID-19 positive family member or friend. These participants were labeled as the positive group and the remaining as the non-positive group. The results suggest that a higher proportion of participants with a COVID-19 positive relative or friend responded positively to all the fourteen questions. However, the only response to “Family practices of disinfection after coming back from outside” was statistically significant, having a p-value of <0.05 with 87.6% of COVID-19 positive patient group responding more positively than 81.9% of COVID-19 negative group.

Independent sample t-tests were conducted to compare clinical and non-clinical categories and positive and non-positive groups. Mean, standard deviation, and p-values were calculated for both comparison groups with a confidence interval of 95%. The results are detailed in Table 4 (Supplementary Material). The difference between the cumulative positive responses to questions related to preventive measures practices was statistically significant between the preclinical and clinical groups with a p-value of 0.02. No significant difference was observed between cumulative responses to questions about preventive measures among the positive and non-positive groups.

## 4 Discussion

Outbreaks of novel infectious pathogens, like coronavirus, with poorly understood outcomes lead to a significant fear and anxiety among the general population [13]. Health professionals as well as medical students, are affected similarly by pandemics like these and are at higher risk of getting infected. Therefore, it is essential to assess the existing preventive behaviors of medical students across the country.

This study assessed the level to which medical students adhered to prevention practices and compared them between the clinical and preclinical year medical students regarding COVID-19. There was an overall sound response, but still, some practices need addressing. A more significant proportion of clinical year students responded positively compared to preclinical year students to questions related to the preventive practices recommended by WHO (86.15% vs. 83.55%). Statistically significant results were observed in responses to “information on preventive measures for COVID-19″ and “tendency to avoid crowded areas, social distancing, and family practices of disinfection.” Moreover, statistically, significant differences also existed in the cumulative positive responses to these questions in the clinical group compared to the preclinical group. The possible reasons for the difference in responses are that since clinical year students have progressed further in their studies and interact with patients daily, they possess more knowledge and are more aware of the appropriate practices against this global pandemic.

According to literature, this is the first nation-wide study that assesses the effect of having a COVID-19 positive family member or friend on practices. There was a higher proportion of positive responses to all fourteen questions in participants with a COVID-19 positive family member or friend. Statistically, a significant difference was noted in responses to “family practices of disinfection on coming back home”. The rationale for the observed differences is that having a COVID positive relative or friend leads to gathering more information about the disease process and therefore improves the preventive behaviors associated with its prevention. As a result, they go an extra mile to disinfect themselves properly on coming back home.

This study revealed that having a COVID-19 positive family member or friend increased the positive responses to preventive behaviors. No similar literature exists establishing this association and therefore provides an opportunity to investigate more into this causal relationship in subsequent studies.

In a study in China, 97.9% of people reported using masks in public compared to 89.2% of the sample. Decreased mask usage may be why China was able to get a timely hold on the spread of the disease compared to Pakistan. One interesting finding was the presence of good respiratory hygiene (i.e., coughing/sneezing in bent elbows), with 92.3% of the study population claiming to do so as compared to 54.9% of the people in China [14].

A study conducted in Jordan showed active effort from participants to acquire information regarding prevention against COVID-19 using valid medical search engines [15]. This, therefore, resulted in better practices related to the disease as compared to this study. Although this study had a higher positive response to COVID-19 related practices as compared to some of the studies conducted previously in Jordan and Middle East regions [16,17], Taghrir et al. [18] conducted similar survey in healthcare workers of middle east, and reported similar positive responses (86.9%) when compared with this study's findings (86.6%).

This study revealed that individuals with the viral disease showed reluctance to seek medical assistance at the right time. 30.3% of the participants refused to disclose their infection status to healthcare professionals if they had symptoms. These findings were similar to a study conducted previously in Jordan where 15.3% wanted to keep their infectious status a secret. Furthermore, 30.7% of them expressed fear regarding diagnostic and therapeutic approaches against COVID-19 in local hospitals [15]. The possible explanation for these findings is the stigma attached to the disease process and lack of awareness in the general population.

In this study, the practice of disinfecting frequently touched surfaces like doorknobs and handles was relatively low, with a positive response seen in only 68.3% of medical students. These similar findings were noted in the study stated above by Khasawneh et al., where 68.8% of participants practiced disinfection of surfaces [15]. These results suggest that there exists an ambiguity among the population about the importance of disinfecting surfaces. This can be explained by the fact that literature doesn't provide concrete evidence about these practices' importance yet.

In this study, the participants with a positive COVID relative were less able to manage family stress than those without a COVID positive close friend/relative.

The appropriate practice of preventative measures was seen in 75.7% of the study population. Although Nour et al. reported a lower percentage of participants performing appropriate practices in his study conducted in Iran [19], Zhong et al. [20] reported a significantly higher proportion of participants in Chinese population(98%) practicing appropriate preventative strategies when compared to this study. Since China is the country where the outbreak started, more awareness about appropriate measures against COVID-19 is expected.

This study has specific strengths and limitations. Based on literature, this is a first nation-wide study in a low- and middle-income country setting that assesses the practices related to COVID-19 among different subgroups of medical students. A large sample size, students from across the country, and non-probability consecutive sampling also ensured better representation of the population. According to WHO and Cronbach's Alpha guidelines, practices were well assessed above 0.7, showing the questionnaire's reliability. This study's limitations included fewer questions (14 Q's) for behavioral assessment due to scarcity of time during the online quiz.

This study described the preventive measures taken by Pakistani medical students to avoid being infected with the novel coronavirus and the areas that need addressing. The stigma of informing the local health authorities and the proper disinfection of surfaces should be encouraged more as they are less practiced. Overall, Pakistani medical students follow the hygiene recommendations of WHO very well.

Medical students in clinical years show better practices regarding prevention against COVID-19 as compared to preclinical year students. Moreover, having a COVID positive family member or friend also improves the understanding and practices. This study, therefore, can pave the way for future interventions targeting less practiced preventive measures and those subsets of health professionals and medical students in whom the understanding and practices about this viral illness are relatively scarce.

## Ethical approval

We obtained an ethical approval from Faisalabad Medical University for conducting our research.

## Please state any sources of funding for your research

None.

## Author contributions

All authors made substantial contribution to this work.

## Registration of research studies

1. Name of the registry:

2. Unique Identifying number or registration ID:

3. Hyperlink to your specific registration (must be publicly accessible and will be checked):

## Guarantor

Shoaib Ahmad.

## Consent

N/A.

## Provenance and peer review

Not commissioned, externally peer reviewed.

## Declaration of competing interest

None.
