# Supplementary material for: Assessment of COVID-19 related preventive measures in medical students across a lower-middle-income country: A cross-sectional study from Pakistan
Source: Ann Med Surg (Lond). 2022 Sep 25;82:104757. doi: 10.1016/j.amsu.2022.104757 (PMC9509292; doi:10.1016/j.amsu.2022.104757)
Supplement: Multimedia component 2 [file mmc2.docx]

## COVID-19 preventive measures in medical students across a lower-middle-income country: A cross-sectional study from Pakistan

**Table 1:** Demographical information of the study participants

| Variables | N (%) |
| --- | --- |
| Age, mean ± S.D. – (year) | 21 ± 4 |
| Gender – no. (%)  Female  Male | 881 (65.6)  461 (34.4) |
| Educational status – no. (%)  MBBS  BDS | 1,308 (97.5)  34 (2.5) |
| Province – no. (%)  Punjab  FATA  Azad Kashmir  KPK  Sindh  Balochistan | 1,129 (84.1)  102 (7.6)  41 (3.1)  29 (2.2)  22 (1.6)  19 (1.4) |
| Class – no. (%)  Clinical  Preclinical | 849 (63.3)  493 (36.7) |

**Table 2:** Responses of preclinical and clinical year students regarding practices of COVID-19 prevention

| Variables | Total population  N = 1,342 | Year of study | | P-value |
| --- | --- | --- | --- | --- |
|  | **Overall, “Yes” responses**  **N (%)** | **Preclinical**  **N (%)** | **Clinical**  **N (%)** |  |
| Practices | | | | |
| 1. Have information on preventive measures | 1,288 (96) | 463 (93.9) | 825 (97.1) | **<0.01** |
| 1. Necessity of self-isolation when having minor symptoms | 1,222 (91.1) | 445 (90.2) | 777 (91.5) | 0.437 |
| 1. Preference to call local health authorities if symptoms appear | 935 (69.7) | 335 (67.9) | 600 (70.6) | 0.296 |
| 1. Updated about the recent COVID information from trusted sources | 1,204 (89.7) | 439 (89.0) | 765 (90.1) | 0.538 |
| 1. Regular handwashing with soap and alcohol rub | 1,293 (96.3) | 476 (96.5) | 817 (96.2) | 0.763 |
| 1. Avoiding crowded areas and staying at home | 1,274 (94.9) | 458 (92.9) | 816 (96.1) | **<0.05** |
| 1. Avoid touching your face after sneezing or coughing | 1,124 (83.8) | 404 (81.9) | 720 (84.8) | 0.171 |
| 1. Regular use of mask on leaving home | 1,197 (89.2) | 433 (87.8) | 764 (89.9) | 0.219 |
| 1. Social distancing (maintain 3 feet distance) | 1,030 (76.8) | 359 (72.8) | 671 (79.0) | **<0.01** |
| 1. Practice good respiratory hygiene | 1,238 (92.3) | 446 (90.4) | 792 (93.2) | 0.063 |
| 1. Frequently disinfect surfaces (doorknobs, handles, etc.) | 916 (68.3) | 332 (67.3) | 584 (68.7) | 0.584 |
| 1. Take measures to boost immunity (Vitamin C, etc.) | 981 (73.1) | 352 (71.3) | 629 (74.4) | 0.284 |
| 1. Family practices of disinfection after coming back from outside | 1,111 (82.8) | 393 (79.7) | 718 (84.5) | **<0.05** |
| 1. Able to manage stress in pandemic | 1,021 (76.1) | 389 (78.9) | 632 (74.4) | 0.065 |

**Table 3**. Assessment of Preventive measures based on COVID-19 positive family members or friends

| Variables |  | ATTITUDE  COVID-19 positive patient in family or close friends | | P-value |
| --- | --- | --- | --- | --- |
|  | **Overall, “Yes” responses**  **N (%)** | **Yes**  **N (%)** | **No**  **N (%)** |  |
| PRACTICES | | | | |
| 1. Have information on preventive measures | 1288 (96) | 205 (97.1) | 1083 (95.7) | 0.342 |
| 1. Necessity of self-isolation when having minor symptoms | 1222 (91.1) | 193 (91.4) | 1029 (90.9) | 0.820 |
| 1. Preference to call local health authorities if symptoms appear | 935 (69.7) | 151 (71.5) | 784 (69.3) | 0.515 |
| 1. Updated about the recent COVID information from trusted sources | 1204 (89.7) | 187 (88.6) | 1017 (89.9) | 0.570 |
|  | | | | |
| 1. Regular handwashing with soap and alcohol rub | 1293 (96.3) | 203 (96.2) | 1090 (96.3) | 0.906 |
| 1. Avoiding crowded areas and staying at home | 1274 (94.9) | 201 (95.2) | 1073 (94.8) | 0.813 |
| 1. Avoid touching your face after sneezing or coughing | 1124 (83.8) | 171 (81.0) | 953 (84.2) | 0.245 |
| 1. Regular use of mask on leaving home | 1197 (89.2) | 189 (89.5) | 1008 (89.1) | 0.847 |
| 1. Social distancing (maintain 3 feet distance) | 1030 (76.8) | 163 (77.2) | 867 (76.6) | 0.851 |
| 1. Practice good respiratory hygiene | 1238 (92.3) | 195 (92.4) | 1043 (92.2) | 0.921 |
| 1. Frequently disinfect surfaces (door knobs, handles, etc.) | 916 (68.3) | 151 (71.5) | 765 (67.6) | 0.261 |
| 1. Take measures to boost immunity (Vitamin C, etc.) | 981 (73.1) | 159 (75.3) | 822 (72.7) | 0.421 |
| 1. Family practices of disinfection after coming back from outside | 1111 (82.8) | 185 (87.6) | 926 (81.9) | **<0.05** |
| 1. Able to manage stress in pandemic | 1021 (76.1) | 151 (71.5) | 870 (76.9) | 0.09 |
| P values are based on chi-squared tests | | | | |

**Table 4:** Cumulative comparison of both groups by t-test

| Variable | N | Preventive measures  (Practices) | Independent sample t-test |
| --- | --- | --- | --- |
|  |  | **Mean ± S.D.** | **P-value** |
| Pre-clinical | 493 | 11.61 ± 2.37 | **0.02**  **(p<0.05)** |
| Clinical | 849 | 11.90 ± 2.28 |  |
| Non-positive group | 1,131 | 11.78 ± 2.38 | 0.64 |
| Positive group | 211 | 11.86 ± 1.94 |  |
